# Resveratrol chemosensitizes HER-2-overexpressing breast cancer cells to docetaxel chemoresistance by inhibiting docetaxel-mediated activation of HER-2–Akt axis

**DOI:** 10.1038/cddiscovery.2015.61

**Published:** 2015-12-07

**Authors:** B S Vinod, H H Nair, V Vijayakurup, A Shabna, S Shah, A Krishna, K S Pillai, S Thankachan, R J Anto

**Affiliations:** 1 Cancer Research Program, Division of Cancer Research, Rajiv Gandhi Centre for Biotechnology, Thiruvananthapuram, Kerala, India

## Abstract

As breast cancer cells often develop chemoresistance, better therapeutic options are in search to circumvent it. Here we demonstrate that human epidermal growth factor receptor-2 (HER-2)-overexpressing breast cancer cells resist docetaxel-induced cytotoxicity by upregulating HER-2 and its activity downstream, through Akt and mitogen-activated protein kinase (MAPK) pathways. We observed that introducing resveratrol as a chemosensitizer in docetaxel chemotherapy blocks upregulation and activation of HER-2 in addition to blocking downstream signaling pathways such as Akt. Resveratrol and docetaxel combination results in the synergistic induction of cell death in HER-2-overexpressing SK-BR-3 cells, whereas introduction of wild-type HER-2 in MDA-MD-231 cells increased the resistance to docetaxel. Dominant-negative HER-2 sensitizes SK-BR-3 cells to docetaxel. Our study identified a new synergistic therapeutic combination that targets HER-2-induced breast cancer resistance and might help to overcome therapeutic resistance during breast cancer therapy. The synergism of docetaxel and resveratrol was maximum in SK-BR-3, which is unique among the cell lines studied, due to its high expression status of HER-2, a receptor known to dictate the signaling environment of breast cancer cells. Docetaxel could further induce HER-2 activity in these cells, which was downregulated on resveratrol treatment. Transfection of DN-HER-2 in SK-BR-3 cells inhibits the synergism as the transfection itself sensitizes these cells to docetaxel, leaving no role for resveratrol, whereas ectopic expression of HER-2 introduces the synergism in MDA-MB-231, the triple-negative cell line, in which the synergism was minimum, attesting the crucial role of HER-2 in suppressing the sensitivity to docetaxel. Single-agent docetaxel induced HER-2-mediated resistance to cell death, which was blocked by resveratrol. Resveratrol also downregulated docetaxel-induced activation of MAPK and Akt, survival signaling pathways downstream of HER-2. In short, this study, for the first time, establishes the role of HER-2–Akt signaling axis in regulating the synergistic effect of docetaxel and resveratrol in breast cancer cells overexpressing HER-2.

## Introduction

Being the most frequently diagnosed female cancer worldwide, breast cancer is always a mystifying puzzle, owing to its highly heterogeneous and complex nature. The molecular intricacies associated with breast cancer raise a unique curiosity among the researchers to unravel the mystery behind the wide contrast in responsiveness to treatments by the different subtypes of breast cancer. The expression status of estrogen receptor (ER), progesterone receptor (PR) and human epidermal growth factor receptor-2 (HER-2/neu) is highly significant in clinical scenario due to the influence of these trios in dictating the response of breast cancer cells to the currently available chemotherapeutic agents.^1,2^ Hormonal receptors are ideal therapeutic targets and tumors overexpressing them usually respond well to hormonal therapy.^3^ On the contrary, the HER-2 overexpression is considered as a clinical dilemma, which often portends tumour aggressiveness and chemotherapeutic resistance in tumor cells. In 20–25% of invasive breast cancers, *HER-2* gene is either overexpressed or amplified.^4^ As an epidermal growth factor receptor (EGFR), HER-2 is not known to bind with any known ligands but can heterodimerize with other related EGFR family members. By doing so, it could recruit various adaptor proteins, which in turn lead to the activation of multiple signal transduction cascades including RAF–MEK–ERK and PI3K/AKT/mTOR pathways.^5^ All these signaling events activated on HER-2 overexpression provide a pro-survival environment in breast cancer cells leading to chemotherapeutic resistance.

Docetaxel, an FDA-approved taxane frequently used for frontline therapeutic treatment of breast cancer, exerts its cytotoxicity by altering the dynamics of tubulin formation in cancer cells.^6^ Although docetaxel is efficient in blocking its target, the general inefficiency of this drug to overcome the survival signals, which get activated in response to its treatment, often leads to chemotherapeutic resistance and even to tumor relapse. Multiple survival mechanisms accounting to the docetaxel resistance include enhanced activity of drug efflux pumps and increased activation of general survival signals.^7^ There are ample evidence regarding the activation of survival signals including the prominent kinase networks such as mitogen-activated protein kinase (MAPK) and PI3K/Akt or the transcription factor such as nuclear factor-*κ*B (NF-*κ*B) and AP-1 in response to docetaxel treatment, which in turn could create a cellular pro-survival environment leading to apoptosis resistance.

Chemosensitization is an attractive strategy to surmount such drawbacks involved in docetaxel treatment. A compound that can efficiently downregulate the survival signals activated by docetaxel can act as a chemosensitizer and can enhance its efficacy. Several pharmacologically safe phytochemicals have been reported to act as potent chemosensitizers in combination with conventional chemotherapeutic drugs.^8,9^ Resveratrol, a natural chemopreventive, is one among them and possesses all attractive traits such as multi-targeting efficacy, pharmacological safety, immediate availability and cost effectiveness, which are required for a classic chemosensitizer.^10–12^ The relationship between HER-2 signaling and taxane resistance are mediated through activation of PI3K/Akt and upregulation of survivin, a factor known to help the tumor cells to avoid taxane toxicity by inducing an early mitotic exit.^13–16^ Similarly, HER-2 is shown to influence the multi-drug efflux pump activation, a crucial factor known to provide resistance against drugs including taxanes, through MAPK–STAT-3 signaling axis.^15,17,18^ Reviewers who meta-analyzed the reports on docetaxel resistance noted a battery of such signaling networks with HER-2 as its focal point, which helps them to relegate this receptor as a governing factor of taxane resistance.^15^ However, the practical attempt to enhance the efficacy of chemotherapeutic agents by blocking HER-2 receptor molecule has so far been not successful as expected.

Here we show that resveratrol as a combination with docetaxel blocks HER-2 expression and its activation in addition to blocking downstream signaling pathways such as Akt. Resveratrol and docetaxel combination results in the synergistic induction of cell death in HER-2-overexpressing SK-BR-3 cells, whereas introduction of wild-type HER-2 in MDA-MD-231 cells increased the resistance to docetaxel. Dominant-negative HER-2 sensitizes SK-BR-3 cells to docetaxel. Our study, for the first time, identified a novel therapeutic combination that targets HER-2-induced breast cancer resistance to induce apoptosis synergistically and might help to overcome therapeutic resistance during breast cancer therapy.

## Results

### Docetaxel and resveratrol exerts synergistic cytotoxic effect in breast cancer cells, while normal immortalized breast epithelial cells are unaffected

**Figure 1:**
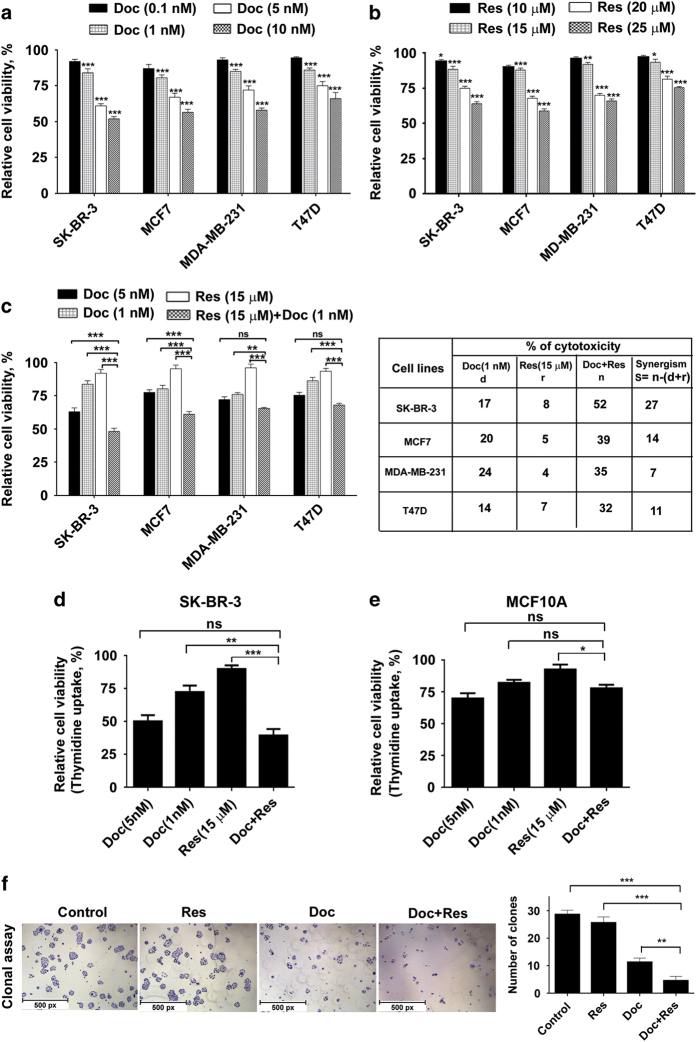
Resveratrol sensitizes breast cancer cells to docetaxel-induced cytotoxicity, while being non-toxic to normal immortalized breast cells. (**a**) Dose-dependent cytotoxicity of docetaxel (0.1–10 nM) on breast cancer cells with varied receptor status. (**b**) Effect of different concentrations of resveratrol (10–25 *μ*M) on different breast cancer cells. (**c**) Effect of docetaxel (1 nM) and resveratrol (15 *μ*M), alone or in combination on different breast cancer cells. Cells (5×10^3^) in triplicates were exposed to the indicated concentrations of the docetaxel for 48 h and subjected to MTT assay. Relative cell viability was determined as percentage absorbance over untreated control. (**d**) Effect of docetaxel and resveratrol, alone or in combination, on SK-BR-3 cells using [^3^H] thymidine incorporation assay. Cells (5×10^3^) in triplicates were exposed to the indicated concentrations of the drugs for 24 h and subjected to [^3^H] thymidine incorporation assay. Relative cell viability was determined as percentage thymidine incorporation over control. (**e**) The combination is non-toxic to MCF10A as assessed by [^3^H] thymidine incorporation assay. (**f**) Inhibitory effect of docetaxel and resveratrol, alone or in combination, on the clonogenic ability of SK-BR-3 cells (scale bar, 1 *μ*m/px).

Cell viability assay was performed to evaluate the cytotoxic effect of docetaxel and resveratrol toward breast cancer cells (SK-BR-3, MCF7, MDA-MB-231 and T47D) with varying receptor status. Both the compounds induced dose-dependent cytotoxicity toward the cell lines tested (Figures 1a and b). Various combinations of docetaxel and resveratrol were evaluated for their cytotoxic effect, where a combination of 15 *μ*M resveratrol and 1 nM docetaxel was found to induce synergic cytotoxicity (Figure 1c), which was maximum in SK-BR-3 and minimum in MDA-MB-231, while being moderate in MCF-7 and T47D. The synergistic response exhibited by different breast cancer cell lines to the combination has been depicted in Figure 1c. The contrast in the synergistic response of SK-BR-3 and MDA-MB-231 was evident in the combinative index (CI) values of the combination. CI of SK-BR-3 ranges from 0.32 to 0.51, which is <1, indicating clear synergism, whereas that of MDA-MB-231 ranges from 0.94 to 1.21, which is ⩽1, indicating additive effect. Hence, SK-BR-3 was selected for further evaluation of the combination and the synergism was confirmed by [^3^H] thymidine incorporation assay (Figure 1d). According to the results, docetaxel and resveratrol in combination exerts cytotoxic effect, which is more or similar to the cytotoxicity induced by five times higher concentration of docetaxel alone, whereas resveratrol alone did not induce a significant cell death (Figures 1c and d). The biological safety of the combination was ensured in normal immortalized breast epithelial cell line, MCF10A by [H]^3^ thymidine incorporation assay (Figure 1e). Furthermore, the combination of resveratrol with docetaxel drastically blocked the clonogenic potential of SK-BR-3 cells (Figure 1f).

### The synergism of docetaxel and resveratrol in SK-BR-3 cells is evidenced by enhancement in apoptosis

**Figure 2:**
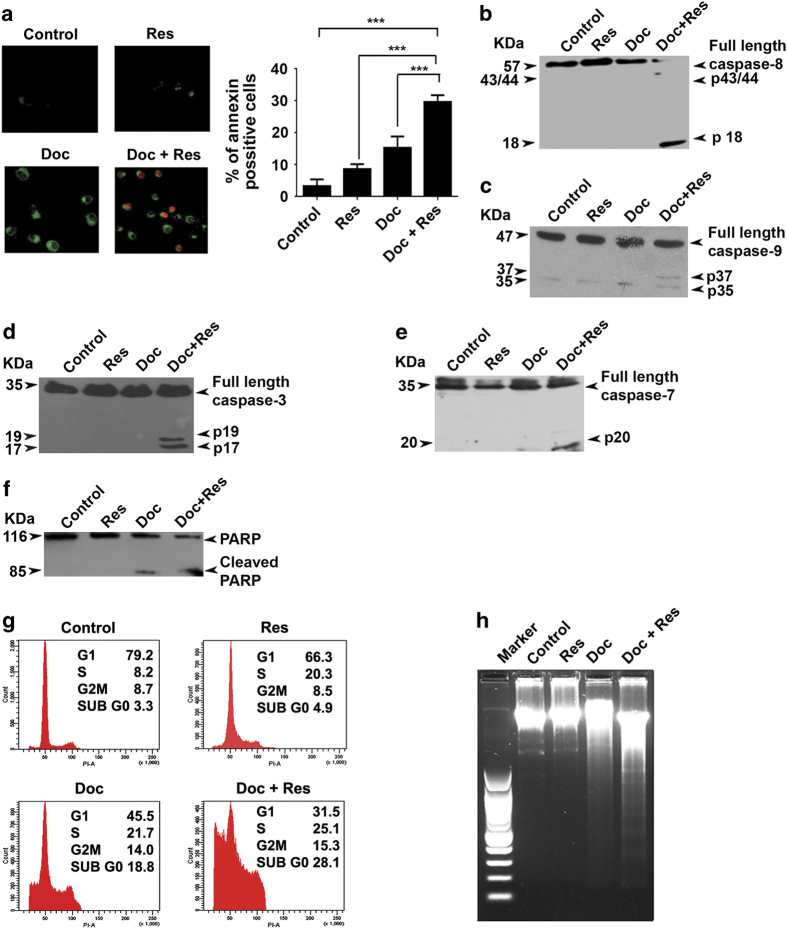
Resveratrol enhances docetaxel-induced apoptosis in SK-BR-3 cells. (**a**) Cells were treated with resveratrol and/or docetaxel for 16 h and stained for Annexin V–propidium iodide (PI) positivity. Annexin V-positive cells in different fields were counted and the average was taken. The green-stained cells are those that have taken only the Annexin V–FITC stain and represent initial stages of apoptosis, and the red-stained cells are those that have taken up both Annexin–FITC and PI, which indicates nuclear membrane damage, and hence represent later stages of apoptosis. Representative histograms indicate percentage of annexin-positive cells. ***P-value ≤0.001.(**b**–**e**) Resveratrol-mediated enhancement of docetaxel-induced caspase activation. Whole cell lysate of cells treated with docetaxel and/or resveratrol for 48 h were blotted against caspase antibodies. (**f**) Resveratrol enhances docetaxel-induced PARP cleavage. Whole-cell extracts were blotted against anti-PARP antibody. (**g**) Effect of docetaxel and resveratrol, alone or in combination, on cell cycle. Cells were collected 48 h post drug treatment, fixed in alcohol, stained with propidium iodide and assayed for DNA content by flow cytometry. (**h**) The effect of docetaxel and/or resveratrol on inter-nucleosomal DNA fragmentation. Cells were treated with docetaxel and/or resveratrol for 48 h, DNA was isolated, run on an agarose gel and visualized. All experiments were repeated at least three times to confirm the reproducibility.

Various apoptotic assays were performed, to confirm the results obtained from the preliminary cytotoxic evaluation of the combination. The results obtained from Annexin V/propidium iodide staining was in concordance with that of MTT assay. SKBR-3 cells treated with the combination exhibited a significant enhancement in externalization of phoshatidyl serine, an early event of apoptosis, compared with that treated with either of these compounds alone (Figure 2a). The combination induced a momentous cleavage of pro-caspase-8 to its active fragment (p18 ) compared with the cells treated with either of the two compounds alone (Figure 2b). The combination also induced the cleavage of procaspase-9, procaspase-3 and procaspase-7 to their active fragments (Figures 2c–e) and a significant enhancement in the cleavage of PARP, the downstream target of caspase cascade (Figure 2f). In addition, treatment with the combination induced a tremendous accumulation of cells in sub-G0 phase (28.1%), confirming the induction of apoptosis by the combination as assessed by PI–FACS analysis. However, resveratrol treatment did not induce a significant enhancement in docetaxel-induced cell cycle arrest (Figure 2g). Moreover, an enhancement in the inter-nucleosomal cleavage of DNA, the biochemical hallmark of apoptosis, was also observed in cells treated with combination (Figure 2h).

### HER-2 has a dominant role in offering resistance to docetaxel

**Figure 3:**
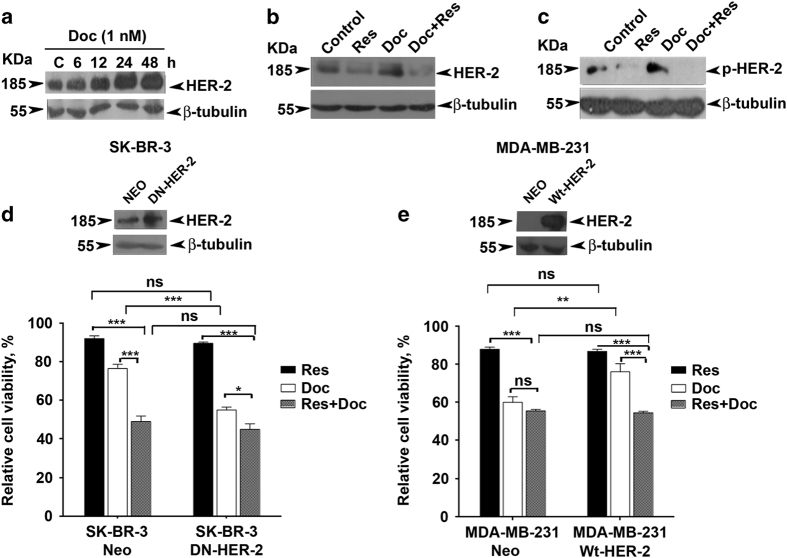
Resveratrol downregulates docetaxel-induced activation of HER-2. (**a** Kinetics of docetaxel (1 nM)-induced activation of HER-2 in SK-BR-3 cells (0–48 h). The whole-cell lysate of docetaxel-treated cells at different time intervals was immunoblotted against HER-2 antibody. (**b** and **c**) Effect of resveratrol treatment on docetaxel-induced overexpression and activation of HER-2 in SK-BR-3 cells. Cells pretreated with resveratrol for 24 h were further exposed to docetaxel and/or resveratrol for another 24 h and immunoblotted against specific antibodies. *β*-Tubulin is used as loading control. (**d**) Effect of HER-2 inhibition on synergism in SK-BR-3 cells. Cells were transiently transfected with vector control and DN-HER-2, respectively. (**e**) Effect of ectopic expression of HER-2 on synergism in MDA-MB-231 cells. Cells were transfected with vector control and WT-HER-2, respectively. The efficacy of transfection was confirmed using western blotting and cell viability was assessed in the transfected cells using MTT assay after treatment with docetaxel and resveratrol alone, or in combination for 48 h in both cases.

As docetaxel achieves its therapeutic efficacy by inhibiting the depolymerization of tubulin and thereby inducing cell cycle arrest, it was surprising to notice that the combination induced a maximum synergistic effect in SK-BR-3 cells among the different breast cancer cell lines studied, while resveratrol did not induce a significant enhancement in docetaxel-induced G2/M arrest in these cells (Figure 2g). This observation logically led us to analyze the difference between the selected cell lines and thus ended up in noting a striking difference in HER-2 expression among them. Although SK-BR-3 is a HER-2-overexpressing cell line, all others express this receptor only at a moderate level.^19^ Hence, we assumed a significant role for HER-2 signaling in the synergism. Interestingly, docetaxel treatment induced further increase in the expression level of HER-2 in SK-BR-3 cells (Figure 3a), which prompted us to evaluate the efficacy of resveratrol in regulating it. Supporting our hypothesis, resveratrol treatment significantly abrogated the basal and docetaxel-induced expression of HER-2 in SK-BR-3 cells (Figure 3b). Concomitantly, the phosphorylation of HER-2, which is an indication of its activity, was also increased on docetaxel treatment and was completely abolished by resveratrol (Figure 3c). To evaluate the role of HER-2 in regulating the synergism, HER-2 signaling was inhibited in SK-BR-3 cells by transfecting DN-HER-2 [K753M] and overexpressed in MDA-MB-231, the triple-negative cell line, by transfecting WT-HER-2, and the synergism was evaluated in these cells and compared with that of vector-transfected cells. Interestingly, the synergism was completely abolished in DN-HER-2-transfected cells, whereas it was persistent in vector-transfected cells (Figure 3d), as the inactivation of HER-2 itself sensitizes the cells to docetaxel and hence there is no significant role for resveratrol as a chemosensitizer. On the contrary, the HER-2-overexpressed MDA-MB-231 cells exhibited resistance to docetaxel compared with its vector-transfected counterparts, whereas its response to resveratrol or to the combination of docetaxel and resveratrol remains unchanged. This, in effect, leads to a significant enhancement of the synergism in HER-2-overexpressed MDA-MB-231 cells, as the introduction of HER-2 contributed to docetaxel resistance, thereby furnishing a chemosensitizing role to resveratrol (Figure 3e). Taken together, these results demonstrate that HER-2 confers resistance to docetaxel and resveratrol overcomes it.

### HER-2 downstream effector Akt is the pivotal molecule that offers resistance to docetaxel and resveratrol downregulates all major survival pathways induced by docetaxel, except NF-*κ*B

**Figure 4:**
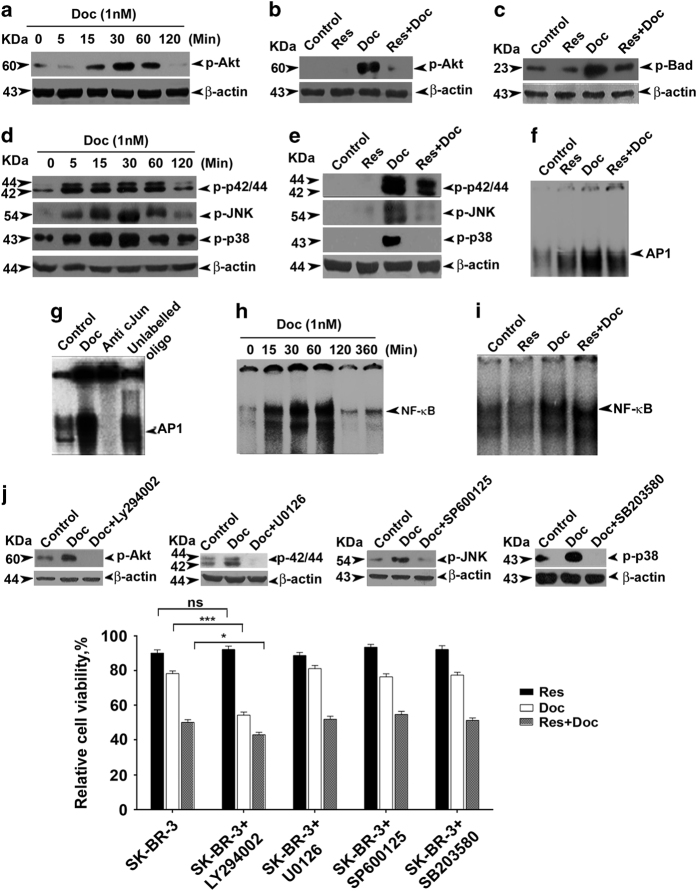
Akt is the regulator of the synergism, although resveratrol downregulates docetaxel-induced upregulation of Akt and MAPK pathways in SK-BR-3 cells. (**a**) Kinetics of docetaxel-induced activation of Akt. Cells were treated with docetaxel for different time intervals (0–2 h). The whole-cell lysate was immunoblotted against phospho-Akt (ser473) antibody. (**b**) Resveratrol-mediated downregulation of docetaxel-induced activation of Akt. Western blot analyses were performed with anti-phospho-Akt (ser473) using whole-cell lysates prepared after 30 min exposure to docetaxel. (**c**) Effect of resveratrol on docetaxel-induced upregulation of phospho-Bad. Western blot analysis was performed against anti-phospho-Bad (ser136). (**d**) Kinetics of activation of MAPKs by docetaxel (0–2 h). The whole-cell lysate was immunoblotted against phospho-specific antibodies of ERK1/2, JNK and p38. (**e**) Resveratrol downregulates docetaxel-induced upregulation of various MAPKs. *β*-Actin was used as loading control in all cases. (**f**) Inhibition of docetaxel-induced activation of AP-1 by resveratrol. Nuclear extracts prepared after exposing the cells to docetaxel and resveratrol, either alone or in combination for a period of 1 h, were assayed for AP-1 activation by EMSA. (**g**) Super-shift analysis using anti-c-jun antibody to indicate band specificity. (**h**) Kinetics of docetaxel-induced activation of NF-*κ*B. Nuclear extracts were prepared after exposing the cells to 1 nM docetaxel for different time intervals (0–3 h) and NF-*κ*B status was assessed by EMSA. (**i**) Individual and combined effects of docetaxel and resveratrol for a period of 30 min on NF-*κ*B activation. NF-*κ*B activation was assayed by EMSA. (**j**) Effect of docetaxel and resveratrol, alone or in combination, in cells treated with Akt and MAPKs inhibitors. Cells (5×10^3^) in triplicates were pretreated with resveratrol, LY294002 (1 *μ*M), U0126 (5 *μ*M), SP600125 (5 *μ*M) and SB203580 (1 *μ*M), followed by docetaxel treatment for 48 h and subjected to MTT assay. Inhibition status of Akt and various MAPKs were shown in inset.

We next examined the role of MAPK, Akt and NF-*κ*B – the survival pathways known to act downstream of HER-2^20–22^ in suppressing the synergism. Docetaxel induces phosphorylation of Akt (Ser 473) as early as 30 min (Figure 4a), which is inhibited by pretreatment of resveratrol (Figure 4b). Concordantly, resveratrol treatment was found downregulating the docetaxel-induced activation of Bad (Ser 136), the immediate downstream target of Akt^22^ (Figure 4c). MAPK signals also maintained a similar response to docetaxel and resveratrol. Docetaxel could induce ERK, JNK and P38 phosphorylation (Figure 4d), which was significantly inhibited on pretreatment of resveratrol (Figure 4e). This pattern of signaling modulation of MAPK in response to docetaxel and resveratrol was also reflected in the nuclear translocation and DNA binding of AP-1, a signaling event downstream of MAPK, as evident in the electrophoretic mobility shift assay (EMSA; Figure 4f). Super-shift assay was conducted to confirm the specificity of AP-1 band (Figure 4g). Docetaxel also induced a transient nuclear translocation of NF-*κ*B (Figure 4h). Surprisingly, resveratrol failed to downregulate this activation of NF-*κ*B (Figure 4i) at the concentration studied (15 *μ*M), although it is a known inhibitor of NF-*κ*B.^23^ As the pattern of signaling modulation exerted by the docetaxel and resveratrol on HER-2 was mirrored in the activation pattern of MAPK and Akt, we hypothesized that any of these HER-2 downstream signaling molecules might act as a major mediator of HER-2-induced resistance to docetaxel.Although inhibition of Akt by LY294002 sensitized SK-BR-3 cells to single-agent docetaxel, inhibition of MAPK pathway by their corresponding inhibitors could not do so, indicating that Akt, but not the MAPK signaling, confers resistance to docetaxel (Figure 4j).

### HER-2 is coupled to Akt axis in regulating the synergism

**Figure 5:**
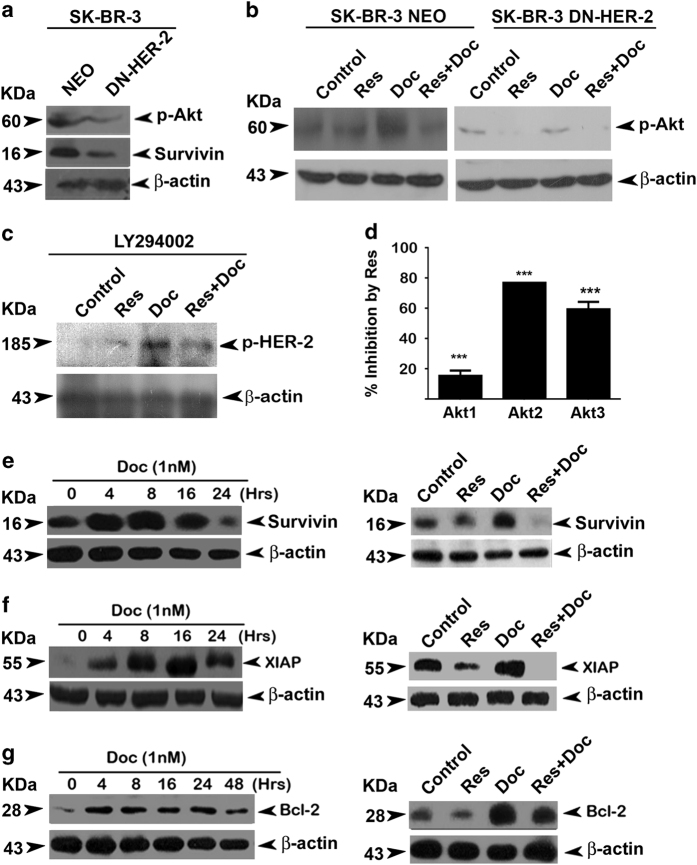
Docetaxel-induced upregulation of XIAP, survivin and Bcl-2 are downregulated by resveratrol in SK-BR-3 cells. (**a**) Effect of HER-2 inhibition on activation of Akt and downstream target survivin. (**b**) Effect of HER-2 inhibition on docetaxel-induced upregulation of phospho-Akt. (**c**) Effect of Akt inhibition on docetaxel-induced activation of HER-2. Western blotting analysis was done using specific antibodies against each molecule and *β*-actin was used as loading control in all cases. (**d**) Resveratrol-mediated inhibition of kinase activity of all three classes of Akt. The study was performed using z-lite biochemical assay platform. (**e**) Kinetics of docetaxel-induced activation of survivin and the inhibition of the same by synergistic combination of resveratrol and docetaxel. The cells were treated with docetaxel for different time intervals (0–24 h) and the whole-cell lysates were immunoblotted against anti-survivin. The cells were pretreated with resveratrol for 24 h followed by docetaxel alone or in combination with resveratrol for 8 h and immunoblotted against survivin antibody. (**f**) Kinetics of docetaxel-induced activation of XIAP and inhibition of the same by synergistic combination of resveratrol and docetaxel. The cells were treated with docetaxel for different time intervals (0–24 h). Western blotting analysis was done against anti-XIAP. The cells were pretreated with resveratrol for 24 h followed by docetaxel alone or in combination with resveratrol for another 16 h and western blot analysis was performed using antibody against anti-XIAP. (**g**) Kinetics of docetaxel-induced activation of Bcl-2 and inhibition of the same by synergistic combination of resveratrol and docetaxel. Cells were treated with of docetaxel for different time intervals (0–48 h). Western blot analysis was performed against anti-Bcl-2. Cells were pretreated with resveratrol followed by combination of resveratrol and docetaxel for 4 h. Whole-cell lysate was immunoblotted using anti-Bcl-2. *β*-Actin was used as loading control in all the experiments.

We have gathered further proof for the existence of HER-2–Akt signaling axis in regulating the synergism. We verified that Akt activity is coupled to HER-2 signaling in SK-BR-3 cells by demonstrating the inhibition of basal Akt signaling in DN-HER-2-transfected cells (Figure 5a). We found that docetaxel could not induce Akt activation in these cells, attesting that arbitration of HER-2 is necessary for docetaxel to induce Akt (Figure 5b). It is interesting to note that Akt inhibition neither blocks HER-2 from being induced in response to docetaxel nor prevent HER-2 from being inhibited in response to the combination of docetaxel and resveratrol, verifying that Akt is downstream of HER-2 (Figure 5c). The independent regulatory role of resveratrol on kinase activity of all three classes of Akt was confirmed by z-lite biochemical assay platform, where isolated Akts (Akt1, Akt2 and Akt3) were screened against resveratrol. Interestingly, the results shows that resveratrol induces maximum inhibition to Akt-2, which is the focus molecule of current study (Figure 5d).

**Figure 6:**
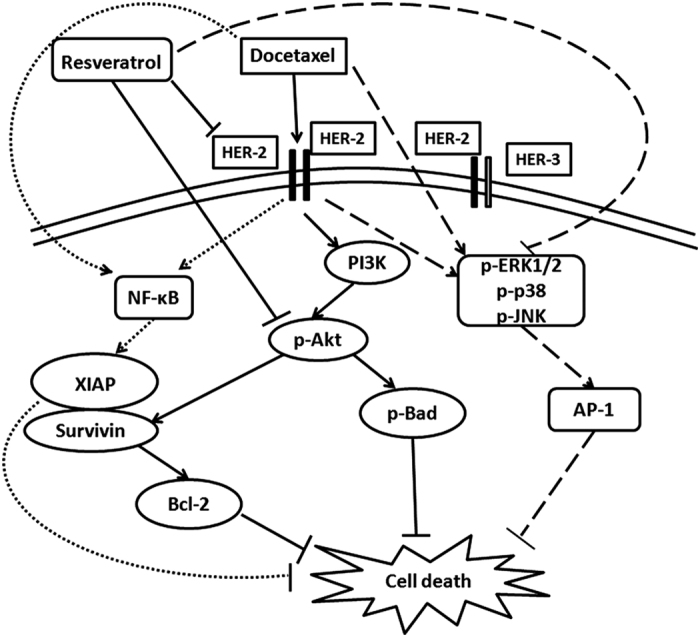
Proposed model for the synergistic effect of docetaxel and resveratrol. Resveratrol downregulates docetaxel-induced upregulation of HER-2, Akt and MAPKs, while that of NF-*κ*B is unaffected. The study postulates that docetaxel-induced upregulation of HER-2–Akt signaling and the downregulation of the same by resveratrol is the main mechanism governing the synergistic effect of docetaxel and resveratrol in HER-2-overexpressing breast cancer cells. Although MAPK pathway does not regulate the synergism, it is getting activated by docetaxel and downregulated by resveratrol. The bold lines indicate the signaling pathways regulating the synergism, whereas the dotted lines represent those that do not have any role in the same.

The response of Akt to docetaxel and resveratrol was also reflected in the expression pattern of the downstream signaling molecules. Resveratrol downregulated docetaxel-induced overexpression of survivin, a pro-survival protein, extensively regulated by Akt^24^ (Figure 5e). Although *XIAP* is generally considered as an NF-*κ*B-dependent gene, it has been shown to be a major target of Akt at post-mitochondrial level.^25^ We found that docetaxel-induced activation of XIAP is downregulated by resveratrol (Figure 5f). Another survival signal, which gets activated in response to the activation of PI3K/Akt pathway is Bcl-2 (Asnaghi, 2004). We observed a time-dependant upregulation of Bcl-2 by docetaxel, which was clearly downregulated by pretreatment with resveratrol (Figure 5g). Taken together, these results demonstrate the efficacy of resveratrol in overcoming docetaxel-induced HER-2 activation and its downstream survival signal Akt, which is pivotal in resveratrol-induced chemosensitization of breast cancer cells to docetaxel. Figure 6 illustrates our findings in a nutshell.

## Discussion

Meta-analysis of the articles describing the anti-tumor efficacy of docetaxel reveals that activation of the general pro-survival signals such as MAPK, NF-*κ*B and Akt in response to docetaxel are the frequently reported reasons for docetaxel chemoresistance. According to the previous studies reporting the mechanism of resveratrol-mediated chemosensitization, the multifaceted efficacy of resveratrol in inhibiting the general pro-survival mechanisms, including the above mentioned signals, forms the very basis of its chemosensitizing property.^8,10,26^ Our results justify the rationale involved in selecting the combination of these drugs, by demonstrating the efficacy of resveratrol in enhancing the signature events of apoptosis such as DNA fragmentation, PARP cleavage and caspase activation induced by docetaxel. However, the correlation observed between the HER-2 expression status of breast cancer cells and their response to the synergistic combination of docetaxel and resveratrol, as described in the Results section, prompted us to approach this study with a perspective based on HER-2 expression status of the breast cancer cells.

Among the growth factor receptors regulating the pro-survival signaling environment in breast cancer cells, HER-2 is deemed important, especially in patient context, because of the influence it exerts on chemoresistance against taxanes. Tumor cells with receptor status profiles similar to that of triple-negative cells (ER^−^, PR^−^ and HER-2^−^) is reported to have a greater sensitivity to taxanes such as paclitaxel than HER-2-amplified cells.^15,27^ SK-BR-3, a cell line that exhibit relatively superior synergism to docetaxel–resveratrol combination, has a higher HER-2 expression status but a lower ER and PR levels, whereas MDA-MB-231, a cell line that exhibits reduced sensitivity to the combination of docetaxel and resveratrol, has a reduced expression of all the three receptors.^19^ It is against this backdrop that HER-2 becomes a factor contributing resistance to docetaxel. The cell lines, SK-BR-3 (ER^−^, PR^−^ and HER-2^+^) and MDA-MB-231(ER^−^, PR^−^ and HER-2^−^), which differ only in their HER-2 status, forms ideal tools for studying its role without the signaling noise from ER and PR receptors.^19^ As major research attempts are devoted so far for improving the chemotherapeutic efficacy by inhibiting HER-2 expression,^28,29^ the evidence regarding the efficacy of resveratrol in antagonizing docetaxel-induced HER-2 upregulation gains significance.

The activity of HER-2 is reported to be mediated through survival signaling pathways such as MAPK and PI3K/Akt.^2,5^ A relatively recent report demonstrates that HER-2 could also positively influence the pro-survival environment of tumor cells through NF-*κ*B mediation.^20^ These signaling molecules, once activated through HER-2, could exert regulations on apoptosis centers and thus mount resistance against stresses by creating a pro-survival environment.^5^ Among them, Akt is the most discussed molecule in connection with HER-2 resistance.^13^ There are reports citing the strong influence of HER-2–Akt signaling axis on tumor resistance against classic chemotherapeutic drugs.^13^ However, the clinical attempts to block this vicious axis using specific HER-2 inhibitors such as Herceptin have met with limited success in clinical trials. One of the reasons pointed out for its failure are the existence of signals capable of activating Akt pathway, independent of HER-2, thus overriding the effect of Herceptin-mediated HER-2 inhibition.^16^ Resveratrol may perhaps have advantage over such quintessential HER-2 inhibitors, as it could block HER-2 and the enzymatic activity of Akt independent of HER-2 as evident from our results. This might help resveratrol to negate all pro-survival signals induced by docetaxel and mediated through Akt. This efficacy of resveratrol in regulating enzymatic activity of Akt independent of HER-2 might be the reason for its synergistic effect with docetaxel in all breast cancer cells screened, irrespective of their HER-2 status.

The extent at which activated Akt exerts its influence over crucial apoptosis regulating molecules should be considered at this context. For instance, survivin and XIAP, members of inhibitors of apoptosis family, are downstream of Akt pathway and are capable of resisting apoptosis induced by classic chemotherapeutic agents including taxanes.^25^ The upregulation of survivin mediated through HER-2–Akt axis has been shown as a reason for the early mitotic exit and thus as a factor influencing the taxane resistance.^15^ Similar is the case of Bcl-2, an anti-apoptotic protein whose activation through Akt helps the cells to maintain mitochondria in an anti-apoptotic signaling mode during a chemotherapeutic insult.^30^ As resveratrol is capable of blocking pro-survival signals mediated through these molecules induced by docetaxel, most probably by blocking HER-2-Akt signaling axis, it is conceivable that blockage of these signals by resveratrol enhances the chemotherapeutic efficacy of docetaxel.

In short, this study, for the first time, illustrates a mechanism-based evidence for the potential utility of resveratrol in overcoming HER-2-mediated chemoresistance of docetaxel in breast cancer cells. However, before proceeding to clinical trials, the proposed mechanism needs further validation using *in vivo* models.

## Materials and Methods

### Cell lines

The breast cancer cell lines SK-BR-3, MCF7, MDA-MB-231 and T47D were purchased from National Centre for Cell Sciences (Pune, India) and the normal immortalized breast epithelial cell line MCF10A (ATCC, Manassas, VA, USA) was a gift from Dr S Sreeja (Rajiv Gandhi Centre for Biotechnology).

### Chemicals

Dulbecco’s modified Eagle’s medium was obtained from Life Technologies (Grand Island, NY, USA); antibodies against caspases, phospho-ERK1/2, phospho-JNK, phospho-p38, Akt, phospho-Akt, phospho-Bad, HER2 and phospho-HER2 were obtained from Cell Signaling (Beverly, MA, USA); and those against c-Jun, survivin, XIAP, Bcl-2 and PARP were purchased from Santa Cruz Biotechnology (Santa Cruz, CA, USA). Resveratrol was purchased from Calbiochem (San Diego, CA, USA). All other chemicals including docetaxel were purchased from Sigma Chemicals (St. Louis, MO, USA).

### Mode of treatment

In all combination treatments, resveratrol (15 *μ*M) was added 24 h before docetaxel (1 nM) treatment unless otherwise mentioned. The DMSO concentration in all experiments, including controls, was ≤0.2%.

### MTT assay

Proliferative/cytotoxic effect of docetaxel and/or resveratrol was determined by MTT assay as described earlier.^31^

### Statistical analysis

The error bars represent ±S.D. of the experiments. For the flow cytometry, data analysis was carried out using the BD FACS Diva software, version 5.0.2 (Becton Dickinson and Company, Franklin Lakes, NJ, USA). The comparison of mean data among multiple groups was analyzed by ANOVA; ***P-values≤0.001, **P-values≤0.01 and *P-values ≤0.05; ns represents nonsignificance.

### Determination of combinatorial effects

The CI was determined as described by Chou and Talalay.^32^ Combinations having CI value <1 were taken as synergistic, those with CI value=1 were taken as additive and those with CI values >1 were taken as antagonistic. The most effective synergistic combination was selected for further studies.

### [^3^H]thymidine incorporation assay

[^3^H]thymidine incorporation assay was performed to assess inhibition of DNA synthesis induced by various drugs as described earlier.^31^

### Clonogenic assay

Clonogenic assay was performed in SK-BR-3 cells treated with required concentrations of resveratrol and docetaxel alone and in combination as descried. Briefly, 500 cells were seeded in 12-well plates and treated with docetaxel (1 nM) and resveratrol (15 *μ*M), alone or in combination. After 72 h, the plates were replaced with fresh medium and incubated for 1 week. The clones developed were fixed in gluteraldehyde and stained using crystal violet. The clones were counted and compared with the control. Colony containing more than ten cells was counted as one clone.^33^

### Western blot analysis

Total protein isolated from cells after indicated treatments were subjected to western blotting as described earlier.^31^

### Annexin-V staining

Phosphatidylserine externalization was observed by staining the cells with fluorescein isothiocyanate-conjugated Annexin-V (Santa Cruz Biotechnology) according to the manufacturer’s instructions and were photomicrographed.

### Cell cycle analysis

The cell cycle analysis was conducted as reported earlier.^31^

### Preparation of nuclear extracts and EMSA assay

EMSA assay was performed to evaluate DNA-binding activity of NF-*κ*B or AP-1 as described earlier.^31^

### Transfection

SK-BR-3 cells were transiently transfected with empty vector and HER2 dominant-negative plasmid, whereas MDA-MB-231 cells were transfected with empty vector and HER2 wild-type plasmids (both plasmids were a kind gift from Dr Mien-Chie Hung, Department of Molecular and Cellular Oncology, MD Anderson Cancer Center, Houston, TX, USA) using Lipofectamine 2000 reagent according to the manufacturer’s protocol (Invitrogen, Life Technologies, Grand Island, NY, USA). Briefly, the diluted DNA sample and the transfection reagent were mixed in 1 : 1 ratio and added to 60–70% confluent cells and incubated for 5–7 h before replacing transfection media with the normal growth media.^31^

## Abbreviations

HER-2: human epidermal growth factor receptor-2
NF-*κ*B: nuclear factor-*κ*B
MAPK: mitogen-activated protein kinase.
